# The ventral capsule and ventral striatum—Stereotactic targets for the management of treatment-resistant depression. A systematic literature review

**DOI:** 10.3389/fpsyt.2023.1100609

**Published:** 2023-10-20

**Authors:** Michał Sobstyl, Marek Prokopienko, Tadeusz Pietras

**Affiliations:** ^1^Department of Neurosurgery, Institute of Psychiatry and Neurology, Warsaw, Poland; ^2^Second Department of Psychiatry, Institute of Psychiatry and Neurology, Warsaw, Poland

**Keywords:** major depressive disorder, nucleus accumbens, deep brain stimulation, treatment-resistant depression, ventral striatum

## Abstract

**Background:**

Deep brain stimulation (DBS) is still an experimental treatment modality for psychiatric disorders including treatment-resistant depression (TRD). There is preliminary evidence that stimulation of brain reward circuit structures including the ventral striatum (VS) may exert an antidepressant effect. The main nucleus of the reward circuit is the nucleus accumbens (NAc). The NAc is a major structure of VS that plays a critical role in reward-seeking behavior, motivation, and addiction.

**Aims:**

This study aimed to review the current studies including randomized clinical trials, open-label trials, and case reports of NAc/VS and VC DBS for TRD in humans.

**Method:**

The literature was reviewed using a medical database—Medical Literature, Analysis, and Retrieval System Online (MEDLINE) on NAc/VS or VC DBS in TRD. The identified studies were assessed based on the patient's characteristics, clinical outcomes, and adverse events related to DBS as well as the stereotactic technique used to guide the implantation of DBS electrodes. The inclusion and exclusion criteria of DBS for TRD were presented and discussed.

**Results:**

The searched literature revealed one case report, three open-label studies (OLS), one multicenter open-label study (mOLS), and two randomized clinical trials (RCTs). There were three additional studies reporting the clinical outcomes in the long term in TRD patients included in the two mentioned RCTs. The total number of patients with TRD treated by NAc/VS or VC is estimated to be 85 individuals worldwide. The response rate to DBS defined as a 50% reduction of postoperative Montgomery-Asberg Depression Rating Scale (MADRS) scores was achieved in 39.8% of the operated patients (range, 23–53%). The remission defined as MADRS scores of < 10 was found in 17.8% after DBS (range, 0–40%). The mean follow-up was 19.7 months (range 3.7–24 months).

**Conclusion:**

The current results of NAc/VS and VC DBS are still limited by a relatively small number of patients treated worldwide. Nevertheless, the results suggest that NAc/VS and VC can be regarded as promising and efficacious targets for DBS, taking into account the response and remission rates among TRD patients with no other treatment option. The adverse events of NAc/VS and VC DBS are reversible due to the adjustment of stimulation parameters. The most common adverse events were hypomanic/manic states, suicidal thoughts/attempts, and suicides. Patients with TRD after NAc/VS and VC DBS should be strictly followed to prevent or diminish these stimulation-induced adverse events.

## Introduction

Major depressive disorder (MDD) is one of the most common psychiatric disorders and a leading cause of disability worldwide (1, 2). There exist several non-invasive, and effective treatment modalities for the management of MDD. The most common conventional treatments are pharmacotherapy, psychotherapy including cognitive-behavioral therapy (CBT), and electroconvulsive therapy (ECT) (3).

The prevalence of depression within the general population is estimated at 3.8% with a higher incidence of 5.0% among adults and 5.7% among adults older than 60 years (4). It is widely accepted that 30% of MDD patients are ultimately diagnosed with TRD. Based on the frequency of reporting in the literature, the most common TRD definition requires a minimum of two prior treatment failures for MDD and confirmation of a prior adequate dose and duration of treatment (4). Patients suffering from TRD more often require hospitalizations and attempt suicide more frequently. It is estimated that 30% of patients with TRD attempt suicide (4). Additionally, patients with TRD show a higher demand for other treatment options such as repetitive transcranial magnetic stimulation (rTMS) and vagus nerve stimulation (VNS) (5, 6). The promising but most invasive treatment modality for TRD patients is deep brain stimulation (DBS). DBS can be the last therapy option for patients who fail to respond to other, less invasive, neuromodulation treatment modalities (7).

The classical symptoms of TRD include depressed mood, decrease in energy, apathy, and anhedonia (7). It is hypothesized that these symptoms may be improved by NAc/VS or VC DBS—the main structures of a reward circuit in humans. These structures incorporating also the ventral part of the anterior limb of the internal capsule (vALIC) were used in the past for ablation and were subsequently replaced by DBS procedures for the treatment of MDD or intractable obsessive–compulsive disorder (OCD) (8). Patients with treatment-resistant OCD stimulated at NAc/VS or VC experienced an improvement in mood and motivation before obsessions and compulsions began to subside (8–10). This finding was the motivation for VC/VS stimulation for TRD (8–10).

The aim of this systematic review was a detailed description of clinical studies regarding DBS of the above mentioned structures. The neuroanatomical connections of NAc/VS and especially NAc are presented. The pivotal role played in the reward system by NAc is presented as a key structure and possible stereotactic target for the neuromodulation of TRD. The safety profile of NAc/VS and VC DBS is also discussed with the most common stimulation-induced adverse events related to these targets.

## The pivotal role of the nucleus accumbens (ventral striatum) in anhedonia and reward processing in depression

A core symptom of MDD or TRD is anhedonia—the inability to experience positive emotions from an activity that was previously associated with reward effects. The most prominent neuroanatomically defined structures of the reward system include the anterior cingulate cortex (AAC), orbitofrontal cortex (OFC), the NAc within VS, and the ventral tegmental area (VTA) (11). It is believed that dysfunction of the reward system can be restored by neuromodulation of the NAc/VS (12).

The NAc is a central structure for processing reward and pleasure information (13). The increases in neuronal activity within NAc and the release of dopamine from the mesolimbic pathway into the NAc are observed during the expectations and experience of rewards (14). This abnormally increased neuronal activity within NAc is also visualized in neuroimaging studies after dextroamphetamine administration, cocaine-induced euphoria, monetary reward, or seeing attractive faces. The administration of dextroamphetamine in patients with MDD produces increased abnormal activity in NAc/VS. These observations highlight the role of NAc in experiencing reward and pleasure and indicate that this region may be dysfunctional in patients with MDD or TRD (15).

The NAc is known to be the interface between the limbic system involved in emotion and motor control. Reciprocal connections of NAc with brain regions involved in emotion processing include ACC, OFC, prefrontal cortex (PFC), and VTA. Reciprocal connections with motor regions encompass dorsal caudate and globus pallidus (16). The NAc is subdivided into limbic and motor subregions known as the NAc shell and NAc core. The shell of the NAc occupies its medial, ventral, and lateral parts, whereas the core occupies its central and dorsal parts (13). The medium spiny neurons in the NAc receive input from both the dopaminergic neurons of the VTA and the glutamatergic neurons of the hippocampus, amygdala, and medial prefrontal cortex. The NAc attains a central position between limbic and mesolimbic dopaminergic structures, basal ganglia, and limbic prefrontal cortices. This central position of the NAc within the VS influences reward-seeking behaviors and reward-related motivational behavior (13, 16).

**Figure 1:**
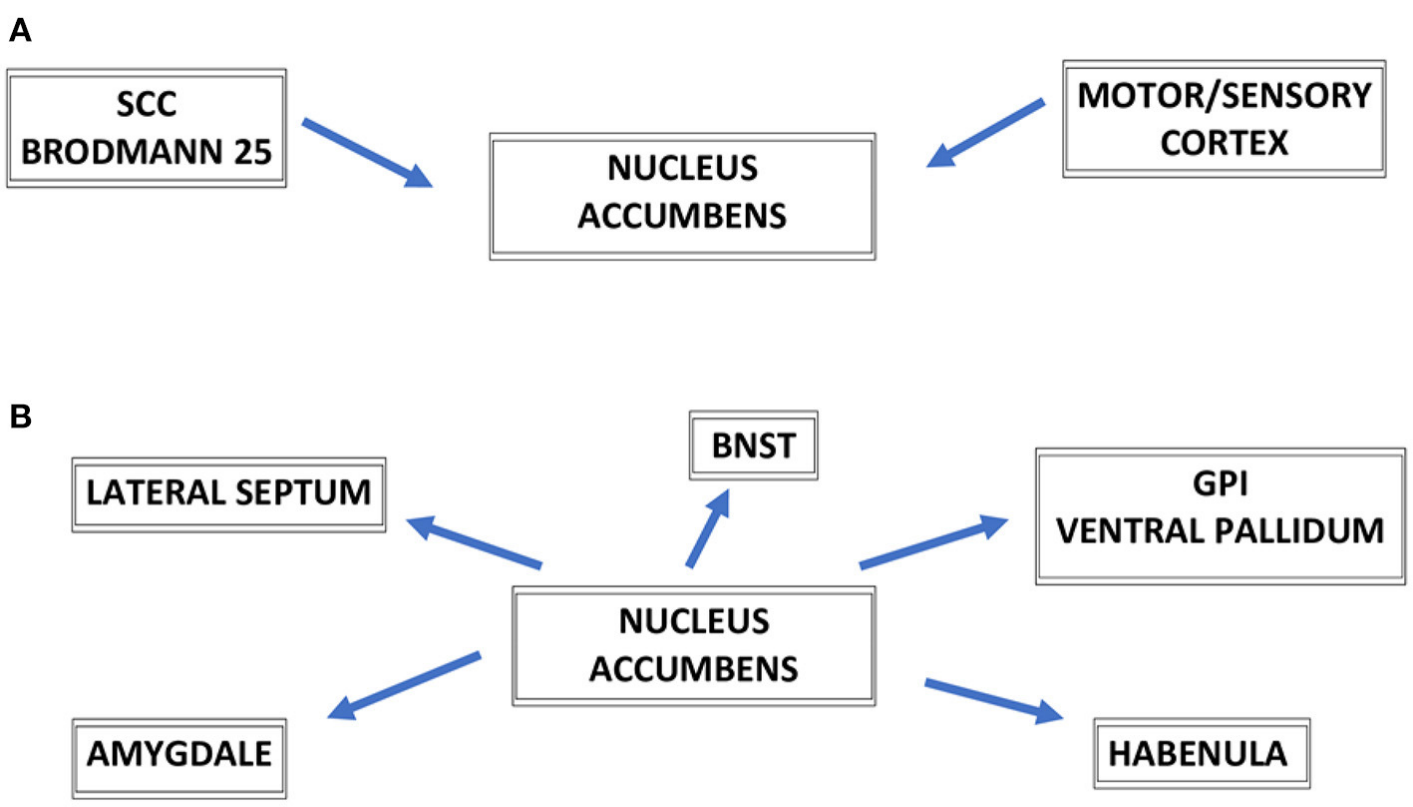
Schematic representation of major connections of nucleus accumbens—NAc, afferent connections to the NAc **(A)**, efferent connections to the NAc **(B)**. SCC, subcallosal cingulate cortex (Brodmann area 25); BNST, bed nucleus of the stria terminalis; GPi, globus pallidus internus; GPi, globus pallidus pars interna.

The NAc has broad reciprocal connections to other brain key regions involved in depression. Moreover, the NAc has indirect projections to brain regions dysfunctional in depression such as the subcallosal cingulate cortex (SCC—Brodman area 25), the medial prefrontal cortex, and the medial pallidum. The VS receives projections from midbrain VTA and brain regions involved in emotion processing such as ACC, OFC, prefrontal cortex (PFC), and amygdala. Moreover, DBS of the NAc with its broad reciprocal connections can modulate neuronal activity in other emotional and motor centers of the brain particularly relevant for the treatment of MDD (11). These broad afferent and efferent connections of NAc are shown in Figure 1. The white matter fibers of the vALIC and the underlying gray matter of VS are collectively referred to as the VC/VS region. DBS of VC/VS has been shown especially effective for refractory obsessive–compulsive disorder (OCD), and also for depressive symptoms accompanying OCD symptomatology (9). Referring to that target as the VC/VS region, the emphasis is placed on the idea that the stereotactic target may be white matter bundles in vALIC/VC not the NAc/VS *per se*. The goal of the stimulation may rely on the modulation of the fibers coursing through this region.

Taking into consideration the model of depression proposed by Mayberg, depression is regarded as an imbalance of the dorsal and ventral components of the limbic cortico-striato-thalamo-cortical (CSTC) mood circuit (17). This model of depression focuses on the assumption that the so-called dorsal compartment of this circuit is hypoactive in contrast to the ventral compartment which is hyperactive. The dorsal component includes both neocortical and midline limbic elements. The main neocortical area of the dorsal component encompasses the dorsolateral prefrontal cortex (dlPFC), dorsal anterior cingulate cortex (dACC), and inferior parietal cortex (iPC). The depressive symptoms including apathy, psychomotor slowing, and decreased ability to perform tests quantifying attention and executive functions are hypothesized to localize mainly to dlPFC, dACC, and iPC.

The ventral component is composed of paralimbic cortical, subcortical, and brainstem regions. The dysfunction of the ventral component is responsible for vegetative and somatic aspects of depression. Sleep, appetite, libido, and endocrine disturbances are caused by the dysfunction of the hypothalamic–pituitary–adrenal axis, insula, subgenual cingulate (Brodmann area 25), and brainstem regions. Both components interact through the so-called rostral compartment with the main functional area of the rostral cingulate cortex. The rostral cingulate cortex regarding its cytoarchitectural characteristics is separated from dorsal and ventral compartments. The rostral compartment plays a major regulatory role in the overall network by facilitating the interactions between the dorsal and ventral compartments. Mayberg's model of depression proposes that the dorsal compartment elements are hypoactive and ventral compartment structures are hyperactive. This model proposes that illness remission occurs when there is inhibition of overactive ventral components regions with concomitant activation of the previously underactive dorsal components regions, especially the dlPFC and dACC. The VS including its main nucleus mainly the NAc has revealed reduced activity in functional imaging which also correlates with the reduced volume of the NAc in structural imaging (18, 19). Both these observations that the function and volume of the NAc are decreased with recurrent MDD episodes have been confirmed additionally by investigating the resting-state functional MRI (rs-fMRI) of the NAc reward network. A study conducted by Ding et al. found that patients with recurrent MDD episodes reveal reduced NAc functional connectivity in the reward network and default mode network (20).

DBS of the NAc/VS by high-frequency stimulation may modulate widespread regional brain regions closely connected to these structures (11). A completely different view is presented by other researchers who claim that the effect of the stimulation of the VC/VS area depends mainly on the stimulation of white matter fibers located in the vALIC rather than the NAc/VS itself (18–20). Some authors still prefer the VS as the functional target itself with its main gray matter structure, mainly the NAc (21, 22).

The therapeutic effects of DBS within NAc/VS or VC extend beyond these target regions. The stimulation effect is not solely related to the structures stimulated but also to the distinct brain regions connected to the CSTC mood circuit dysfunctional in depression (23). DBS may affect not only NAc itself but the co-stimulation of white matter axons running through the vALIC may also occur (23). Depending on the relatively high stimulation settings of NAc/VS or VC DBS, it is likely that other neighboring gray matter structures such as the bed nucleus of stria terminalis (BNST) may also be stimulated (24). The therapeutic effect of NAc/VS and VC DBS on depressive symptoms can be answered by analyzing patient-specific anatomy in regard to the exact location of the DBS electrode with the detailed visualization of the volume of tissue activated (VTA) (25, 26). Functional neuroimaging studies have shed light that NAc/VS or VC DBS exert widespread activity changes in the brain areas dysfunctional in depression (26).

## Material and method for searching of NAc/VS and VC DBS studies for TRD (inclusion and exclusion criteria)

A systematic literature search for the publications regarding DBS in MDD/TRD was conducted spanning the time period from January 2008 to December 2021. The search algorithm included the following keywords: deep brain stimulation, ventral striatum, ventral capsule, nucleus accumbens, anterior limb of the internal capsule, and treatment-resistant depression. The following electronic databases were consulted: Medical Literature, Analysis, and Retrieval System Online (MEDLINE), and Cochrane Central Register of Controlled Trials (CEN-TRAL). The search algorithm followed the PRISMA guidelines (27). Only research articles published in English were considered. The research articles were restricted to clinical studies involving only humans. No limitations were made regarding the study design as well as case reports and case series were included in the present review. A placebo effect is prominent for functional neurosurgical procedures in psychiatric disorders, so the inclusion criterion was a minimum postoperative follow-up period of 3 months.

**Figure 2:**
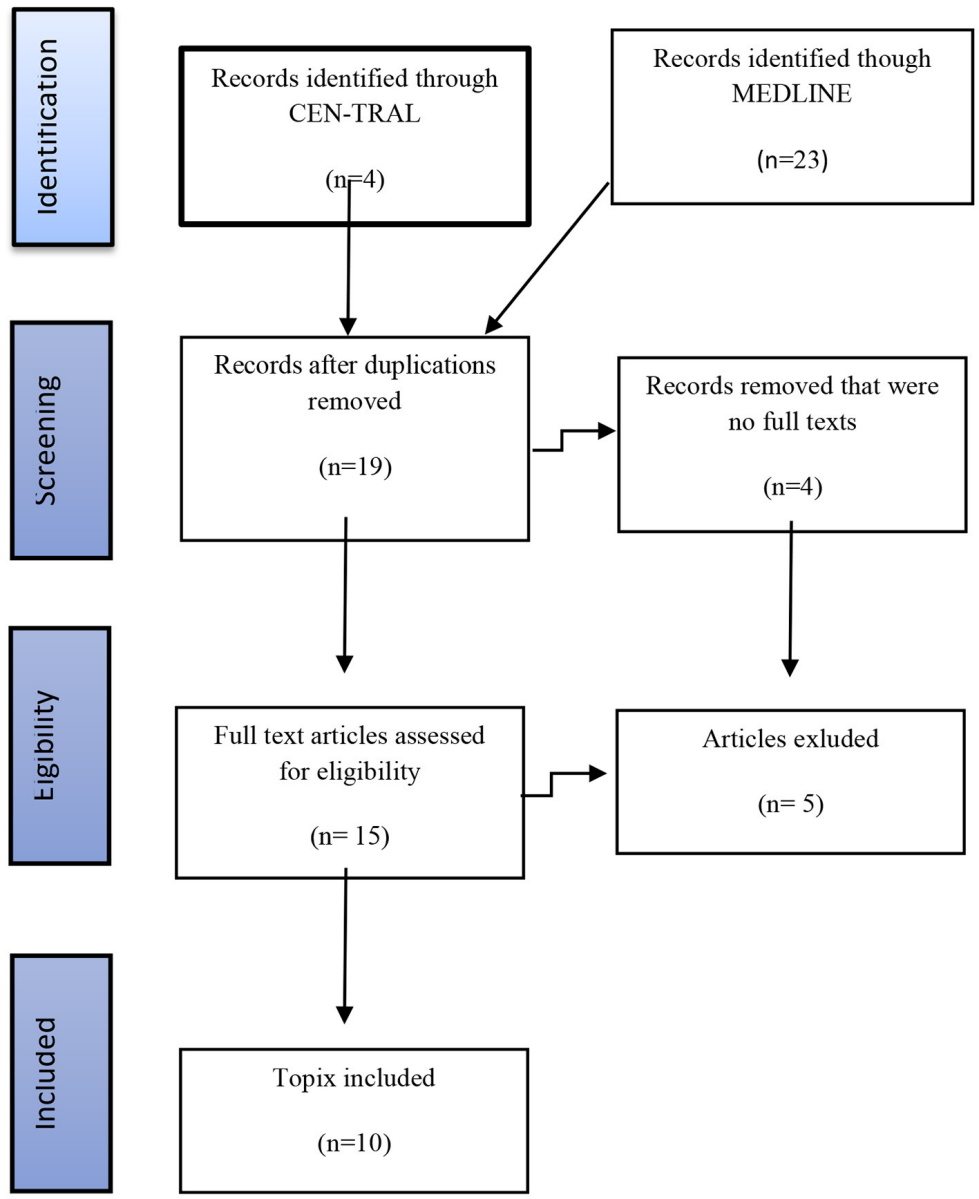
Figure chart illustrating the selection of articles.

The exclusion criteria included animal studies, studies that included treatment of TRD without DBS, preclinical studies, review articles, and letters to the editor. The exclusion criteria included articles describing patient populations other than those with TRD and reports that mainly dealt with the aspects related to the surgical technique. The chart flow showing the search strategy and the final studies selected for the detailed analysis is presented in Figure 2. The search using these two databases and the above mentioned keywords has yielded 27 articles. Using the inclusion and exclusion criteria listed above, we identified 10 articles suitable for further analysis included and discussed below.

## Inclusion and exclusion criteria for NAc/VS and VC DBS for TRD

DBS is the most invasive treatment for TRD. In most studies, the prerequisite is the confirmation of severity, chronicity, disability, and pharmacological refractoriness in patients diagnosed with TRD (21, 28–40). The first step is a correct clinical diagnosis of depression. This disorder is heterogeneous, and diagnostic criteria can be met by combinations of clinical symptoms. The depressive symptoms occur in conjunction with unique social and environmental stressors that work in a dysfunctional manner (21, 29, 30). The prerequisite of an inclusion criterion is the severity of the depressive symptoms scored on validated scales such as the Hamilton Depression Rating Scale (HDRS). On the HDRS, severe depression is scored at least or more than 20 points (21, 29, 30). Social functioning is also profoundly affected by depressive symptoms and scored less than 45 on the Global Assessment of Functioning Scale (21, 29, 30). The history of at least four episodes of major depression or a depression episode of the duration of more than 2 years constitutes the inclusion criteria. A time span of at least 5 years after the first episode of major depression is required for a patient to be considered for a DBS procedure (29–31, 33, 40).

The next step is the establishment of pharmacological resistance. The failure to respond to adequate trials of primary antidepressants from at least three different classes is the prerequisite to considering a patient to be pharmaco-resistant. These trials of primary antidepressants usually last more than 5 weeks at the maximum recommended or tolerated dose. The augmentation period of more than 3 weeks duration to a primary antidepressant at the recommended or tolerated doses is also mandatory (29, 30, 33, 34). At least two augmentation drugs include lithium agents, triiodothyronine, stimulants, antipsychotics, anticonvulsants, buspirone, or secondary antidepressants. The patients are on stable antidepressant drugs for at least 6 weeks before entry into the study (31–33).

The next step in quantification process for DBS in TRD patients is the establishment the refractoriness to the electroconvulsive therapy (ECT). At least six bitemporal ECT treatments are prescribed and will be performed before the qualification process for DDS (29, 30, 33–35). Individual psychotherapy is attempted with at least 20 sessions provided by an experienced psychotherapist (21, 29, 30, 36–39, 41). Written informed consent and the willingness for scheduled postoperative programming sessions are very important to optimize the therapeutic effect (29, 30, 36–39, 41).

The exclusion criteria are to be strictly followed to prevent referring patients with severe comorbid psychiatric disorders that may complicate DBS therapy in this vulnerable group of patients (21, 22, 29, 30, 36, 38). A positive history of severe personality disorder is a contraindication for a DBS procedure in TRD patients (29, 30). The current or non-affective psychotic disorder also constitutes an exclusion criterion in most studies (29, 30). Active substance abuse disorder or remittent addiction (aside from nicotine) constitutes contraindications for DBS surgery in TRD patients (29, 30, 36–39). Patients with brain tumors, vascular malformation, and enlarge ventricular system suggestive of normal pressure hydrocephalus seen in preoperative magnetic resonance imaging studies are usually excluded from DBS procedures for psychiatric disorders (21, 29, 30, 36–39, 41). Patients with severe medical comorbidities who are unsuitable to undergo complicated and usually long-lasting DBS surgery are also excluded (21, 29, 30, 36–39, 41).

The above mentioned inclusion and exclusion criteria can guide the selection of appropriate candidates for TRD DBS. Nevertheless, the selection process is very time consuming and requires strict care from a treating psychiatrist before, during, and after DBS for TRD. The patients qualified for DBS surgery need to be seen at regular intervals for several months for depressive symptoms changes, despite the optimized pharmacotherapy, ECT, and psychotherapy and to ensure that the inclusion criteria are met (21, 29, 30, 36–38). The patients and their family members have to be informed about the realistic expectations for DBS therapy (21, 29, 30, 36–38). In addition, the lack of neurobiological markers of psychiatric disorders coupled with no symptom-specific prediction hinders the selection process. Meeting the inclusion and exclusion criteria and a time-consuming qualification process are responsible for a still small number of TRD patients who undergo DBS procedures worldwide (21, 22, 29–31, 36–40).

## Open-label studies of NAc/VS and VC DBS for TRD

**Figure 3:**
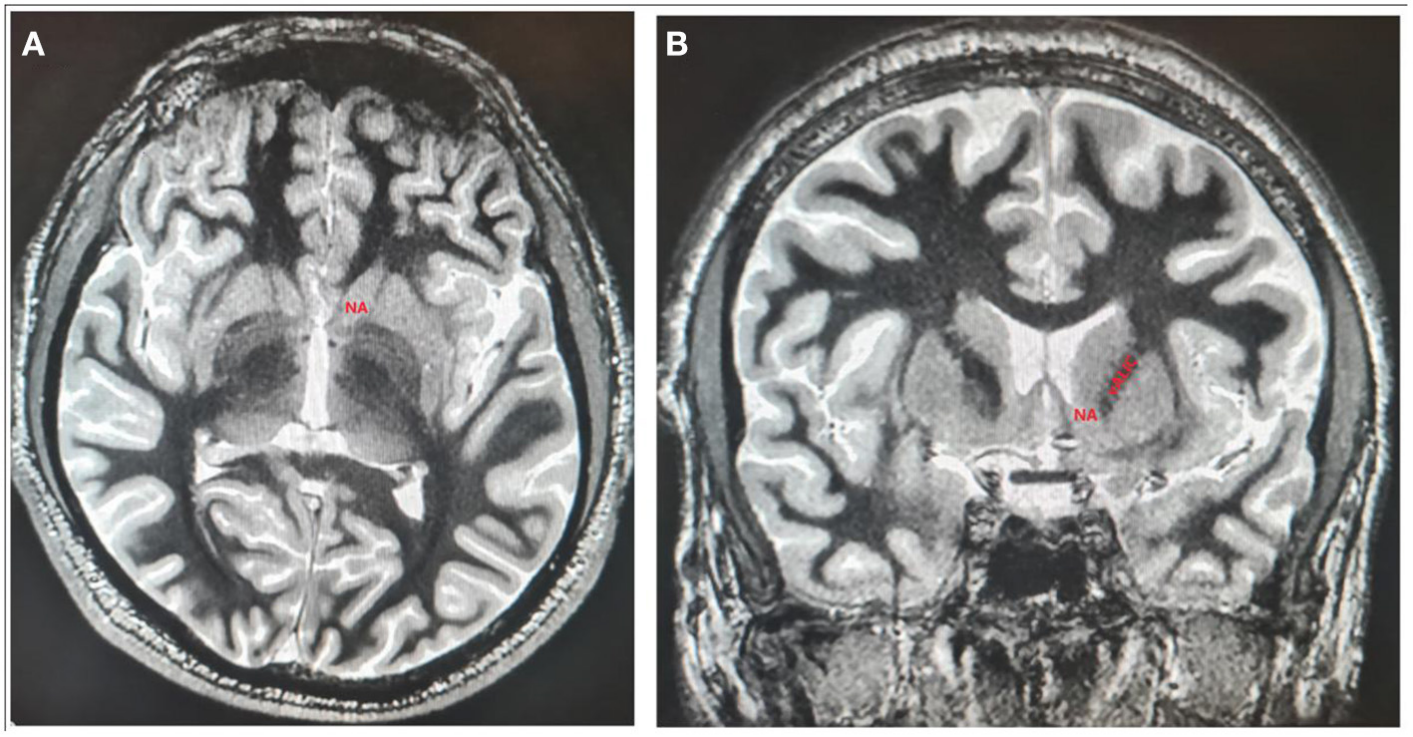
The localization of NAc and vALIC presented on axial **(A)** and coronal **(B)** sections of 3D T1-weighted imaging white/matter null sequence.

The clinical outcomes as well as the adverse events related to DBS of these open-label studies and RCTs of DBS for TRD are presented in Table 1. The stimulation parameters, a mode of stimulation related to NAc/VS and VC DBS are presented in Table 2. The first clinical report including three patients with TRD undergoing NAc DBS was published in 2008 (21). In that trial, the depressive symptoms were evaluated using the HDRS and MADRS scales. The baseline HRDS was 33.7 (±3.8) and the baseline MARDS was 35.7 (±2.9), indicating an extremely severe level of depression (21). A week after bilateral NAc DBS depressive scores dropped to 19.7 (±6.7) and 24.7 (±6.7) (21). After the first week without the stimulation, the scores increased again to 24.7 (±3), and 33.3 (±9.7). The scores in the stimulation off phase did not differ from the baseline scores. This study was further continued in a double-blind manner regarding the stimulation status over the course of several weeks (21). The authors of that study quantified the effects of stimulation on clinical ratings with different stimulation parameters (21). The authors found a negative correlation (increased stimulation led to decreased depression ratings) in all patients for HDRS and MADRS scores. Additionally, the symptomatic worsening during the sham stimulation phase required reinitiation of stimulation in two patients prior to a 4-week blinded placebo period. The study lasted only a few months, so the stability and durability of the response could not be proved (21). This first open-label study has shown that the clinical effect of NAc DBS on depressive symptoms is quick and the effects discontinue rapidly when the patients enter the sham stimulation period (21) (Figure 3).

**Table 1:** Case reports, open-label studies, and randomized clinical trials regarding the clinical safety and efficacy of ventral capsule/ventral striatum and nucleus accumbens deep brain stimulation for treatment-resistant depression presented in chronological order.

**References**	***N***	**Stereotactic target**	**Study design**	**Follow-up in months**	**Response rate at the last follow-up**	**Remission rate at the last follow-up**	**Adverse events**
Schleapfer et al. (21)	3	NAc	OLS	1.4–5.1	33%	0	NR
Malone et al. (31)	15 (14 patients TRD, one patient bipolar disorder)	VC/VS	Multicenter OLS	6–51 (mean 23.5)	53%	40%	Two cases of hypomania One patient with bipolar I disorder experienced hypomanic episode that resolved after stimulation adjustment One DBS lead fracture, One occipital pain requiring the extension revision
Bewernick et al. (29)	10	NAc	OLS	12	50%	30%	One suicide attempt One suicide
Bewernick et al. (30)	11	NAc	OLS	12–48	45%	9%	One suicide attempt One suicide One implant infection and explanation
Sousa et al. (32)	1	NAc	Case report	5	100%	100%	Hypomania/mania
Dougherty et al. (36)	30	VC/VS	CRT	24 maximal follow-up 45	23%	20%	1 Eight patients with worsening depression Five patients with suicidal ideation Five patients with implant infection Four patients with suicide attempts One committed suicide not related to stimulation (non-responder) Three DBS lead revisions
Bergfeld et al. (37)	25	vALIC	CRT	12	40% (10 patients more or 50% reduction of HAM-D-17) 24% (six patients' partial response)	20% (five patients)	One hemorrhage in the supplementary motor area. Four patients had five suicide attempts Two patients with suicidal ideation Two patients died (one suicide and one euthanasia) Two patients with transient symptoms of mania One patient with hypomania Stimulation-induced adverse events in two or more patients blurred vision, sleep disturbances, and disinhibition.
Wal et al. (38)	21	vALIC	Open-label maintenance phase of RCT	24	44.4%	NR	Four instances of adverse events in three patients not related to stimulation (one suicide attempt)
Hitti et al. (39)	8	VC/VS	Open-label design of long-term follow-up phase of RCT	(7.8 ± 4.3 years for the entire cohort)	50%	25%	Five patients with hypomania 5 pts 62.5% One patient with mania 12.5% Recurrent mania, thereafter suicide Two infections 25% requiring electrode and IPG removal and replacement One lead adapters replacement
Bergfeld et al. (41)	19	vALIC	RCT open-label design of long-term follow-up phase of RCT	7.7 ± 1.5 years	44 %	NR	One suicide attempt, one suicidal ideation not related to DBS therapy, and one case of extension cable damage subsequently exchanged

OLS, open-label studies; RCTs, randomized clinical trials; NAc, nucleus accumbens; VC/VS, ventral capsule/ventral striatum; vALIC, ventral anterior limb of the internal capsule; DBS, deep brain stimulation; NR, not reported.

**Table 2:** Stereotactic coordinates of targets in ventral capsule/ventral striatum region including nucleus accumbens for the treatment depression presented in chronological order.

**References**	***N***	**Stereotactic target**	**Stereotactic coordinates related to the anterior commissure (AC)**	**DBS lead type and DBS hardware used**	**Voltage (V)**	**Pulse width (μs)**	**Frequency (Hz)**	**Stimulation mode**
Schleapfer et al. (21)	3	NAc	NR	Medtronic 3,387 leads.	0–5	90	145	Monopolar
Malone et al. (31)	15	VC/VS	X = 6–7 mm lateral to the midline Y = 1–2 mm anterior to posterior border of AC Z = 3–4 inferior to the intercommissural line	13 patients Medronic 3,387 IES lead two patients 3,387 leads Soletra or Kinetra	6.7	90–210	100–130	0, 1 cathodes IPG or three configurated as anodes
Bewernick et al. (29)	10	NAcc	Coordinates of lowest contact X = 7.5 lateral Y = 1.5 anterior Z = 4 mm inferior to the upper front edge of AC.	Medtronic 3,387 leads.	1.5–10	60–210	100–150	Monopolar/bipolar
Bewernick et al. (30)	11	NAcc	Coordinates of lowest contact X = 7.5 lateral Y = 1.5 anterior Z = 4 mm inferior to the upper front edge of AC.	Medtronic 3,387 leads.	Start at 2.0 1.5–10	60–210	100–150	Monopolar/bipolar
Sousa et al. (32)	1	NAc	NR	NR	4.2	150	150	Bilateral bipolar configurations
Dougherty et al. (36)	30	VC/VS	X = 5–10 lateral to the midline Y = 0–5 mm anterior to posterior border of AC Z = 1–5 inferior to the intercommissural line	Medtronic 3,391 leads	Usually below 8 Volt	90 or 210	130	Mostly bipolar configurations
Bergfeld et al. (37)	25	vALIC	X = 7 mm lateral to the midline Y= 3 mm anterior to the anterior border of AC Z= 4 mm below the intercommissural line	Medtronic 3,389 leads Activa PC Deepest contact in Nac and three others in vALIC	2.5–6.0	90	130 or 180	0 or 1 contact used as cathodes
Wal et al. (38)	21	vALIC	X = 7 mm lateral to the midline Y= 3 mm anterior to the anterior border of AC Z= 4 mm below the intercommissural line	Medtronic 3,389 leads Activa PC Deepest contact in Nac and three others in vALIC	2.5–6.0	90	130 or 180	0 or 1 contact used as cathodes
Hitti et al. (39)	8	VC/VS	X = 5–10 lateral to the midline Y = 0–5 mm anterior to posterior border of AC Z = 1–5 inferior to the intercommissural line	Medtronic 3,391 leads	4.0–10.5 The average voltage was 7.2 ± 2.6	210 (80% patients) 90 (20% of patients)	130	Bipolar stimulation (60% of patients) Monopolar (40 % of patients)
Bergfeld et al. (41)	19	vALIC	X = 7 mm lateral to the midline Y= 3 mm anterior to the anterior border of AC Z= 4 mm below the intercommissural line	Medtronic 3,389 leads Activa PC Deepest contact in Nac and 3 others in vALIC	2.5–6.0	90	130 or 180	0 or 1 contacts used as cathodes

The deep brain stimulation hardware and stimulation settings are also presented. NAc, nucleus accumbens; VC/VS, ventral capsule/ventral stiatum; vALIC, ventral anterior limb of internal capsule; DBS, deep brain stimulation.

Based on the results of this study, Bewernick et al. performed an open-label study of 10 patients, who were followed for 12 months (29). The primary outcome measure was antidepressant response defined as a 50% reduction of depressive symptoms severity assessed by the 28-item HDRS or remission defined as an HDRS score of < 10 (29). At 1 year follow-up, 50% of patients were rated as responders and 30% met the criteria for remission (29). Interestingly, the ratings of anxiety evaluated by the Hamilton Anxiety Scale secondary measure in this study were also reduced, which correlated with the increased levels of professional and individual activities (29). The reduction of anxiety scores was more pronounced in responders than in non-responders. Patients in this study underwent PET examination at 6 months after NAc DBS, which revealed significantly reduced metabolism in the amygdala only in responders when compared to non-responders (29). The authors of this study published their long-term results up to 4 years in a subsequent study and found that the antidepressant effect remained stable (30).

The first multicenter open-label study of VC/VS for TRD was presented by Malone et al. in 2009 (31). This study included 15 patients with TRD. The maximal response was observed after 3 months of stimulation, and a 40% response was noted at 6 months. At the last follow-up (mean of 23.5 months), the response rate was 53% and the remission rate was 40% (31). The same year Sousa et al. described a 39-year-old patient who developed panic attacks after NAc DBS for the treatment of refractory obsessive–compulsive and bipolar disorder (32). The main indication for DBS in this patient seemed severe OCD symptoms with comorbid bipolar depression. The follow-up in this case was relatively short reaching 5 months. The OCD and depressive symptoms were well-controlled until the sudden appearance of severe panic attacks. The authors of this case report point to the fact that panic attacks in this particular case were evoked by the stimulation of the most ventral DBS lead contacts. The stimulation at the most ventral contacts (contact 0) may cause panic attacks probably by activation of the amygdala, thus evoking the experience of panic. Shapira et al. and Okun et al. observed the same panic attacks only by the stimulation of the most ventral contacts located next to NAc (33, 34). The panic attacks may have occurred because of the role of NAc as an interface for limbic projections from the amygdala, hippocampus, and cingulate cortex, which receives input from dopaminergic-containing nuclei while maintaining the behavioral and affective changes induced by DBS (35). Additionally, the patient's comorbid bipolar disorder could have facilitated such uncontrollable panic attacks (32).

## Randomized clinical trials of NAc/VS and vALIC (VC) DBS for TRD with subsequent long-term follow-up trials

The above mentioned encouraging results led to the first RCT of VC/VS DBS for TRD (36, 37). This randomized, double-blind, sham stimulation-controlled, multicenter, prospective, parallel design study (Reclaim study) published in 2015 by Dougherty et al. included 30 patients with TRD treated by bilateral VC/VS DBS (36). This study included a 16-week randomized, double-blind sham-controlled phase with a subsequent open-label phase that continued for 2 years (36). The primary outcome measure in this RCT was based on MADRS scores with a response defined as at a least 50% postoperative decrease in MADRS scores. All 30 patients underwent randomization and entered a 16-week blinded-treatment phase (36). The results of the blinded phase failed to show the efficacy of VC/VS DBS for the treatment of TRD. In the active group, three patients out of 15 were responders, compared to the control group where two patients out of 14 were qualified as responders. The baseline MADRS score in the active group was 37.7 (±4.4) and dropped to 29.7 (±12.6), and the baseline MADRS score in the control was 36.4 (±3.3) and dropped to 27.4 (±10.4). These MADRS scores reflect an 8.0% decrease in the active group and a 9.1% decrease in the control group when compared to the baseline MARDS scores (36).

In the open-label continuation phase, the response rates were 20, 26.7, and 23.3% at 12, 18, and 24 months, respectively. Due to the disappointing results, the study was halted after 30 individuals were included (36). Despite these relatively not encouraging effects reflected by a decrease in MADRS scores, 26 out of 30 patients were selected to continue DBS stimulation after 24 months and tolerated the procedure well (36).

A recently published study by Hitti et al. summarizes a decade-long follow-up of eight TRD patients treated by VC/VS DBS as a part of the Reclaim clinical trial published in 2015 by Dougherty et al. (39). At the mean follow-up of 11.0 ± 0.4 years, the response (>50% improvement of MADRS scores) and remission (MADRS score < 10) rates were 50 and 25%, respectively. At the last follow-up (mean 7.8 ± 4.3 years), the mean improvement in MADRS scores was 44.9 ± 42.7%. This study has confirmed the long-term effectiveness of VC/VS DBS in patients with TRD (39). The authors conclude that for a TRD, with no compelling treatment options, the response and remission rates are encouraging (39). In the authors' opinion, the full effect of DBS on TRD symptomatology may not be seen up to even 6 or 7 years (39). The effects of VC/VS DBS were visible at 7 years in four patients. This phenomenon was driven in the author's opinion partly by the fluctuations in some patients' disease severity but other patients, however, did not achieve full benefit until after 5–6 years of stimulation (39). The authors of this study conclude that a sufficient follow-up counted in years is mandatory to assess the effects of DBS on a patient's depressive symptoms and functioning (39). However, it cannot be ruled out, as Hitti et al. claim, that the natural course of TRD, as well as spontaneous remissions of TRD, may affect the results in long-term follow-up (39).

Regarding the stimulation parameters used in the Reclaim study, the initial stimulation settings were very flexible and high, especially the pulse width and initial voltage (36). Adapting to high stimulation settings resulted in a very fast depletion of the implanted Kinetra IPGs which were subsequently replaced by Activa RC rechargeable IPG. The average lifespan of Kinetra IPG was 1.2 ± 0.9 years vs. 7.7 ± 0.2 years when rechargeable Activa RC was implanted. This information is very practical and reduces the costs related to replacements and possible DBS hardware infections when non-rechargeable IPGs have a short lifespan (36, 37).

Another RCT for TRD published in 2016 by Bergfeld et al. had a completely different study design when compared to Doughert et al.'s RCT of VC/VS DBS for TRD (37). This RCT began with an open-label optimization period lasting 52 weeks with a subsequent 6-week duration randomized sham-controlled phase (37). The stereotactic target was vALIC (37). The primary outcome measure was the score of the 17-item HDRS (Hamilton Depression Rating Scale) at the crossover phase between the active and sham groups (37). At 12 months of the open-label optimization period, vALIC DBS resulted in a significant reduction of depressive symptoms in 10 patients (40 %), and 15 individuals were regarded as non-responders (37). In total, 16 patients (nine responders and seven non-responders) entered the sham-controlled crossover phase, where the stimulation was switched off in half of the patients at any given time. During active DBS, patients scored significantly lower on the HDRS scale (13.6 scores) than during sham DBS (23.1 scores). This difference in the active vs. sham stimulation group reached 9.5 scores on the HDRS (37). DBS discontinuation worsened the depressive symptoms in responders, but not in non-responders (37). This study was the first to meet its primary prospective success criterion (37). Further observation of the patients presented by Bergfeld et al. in the maintenance period (2 years after DBS surgery) was provided by Wal et al. (38). Of the 25 patients treated with DBS, 21 entered and 18 patients completed the maintenance phase (38). During the maintenance phase of this study, the severity of HAM-D-17 and MADRS scores did not further change in responders (38). Non-responders did not improve during the maintenance phase (38). Interestingly, the subjective symptoms assessed by the self-reported Inventory of Depressive Symptomatology (IDS-SR) significantly improved between 1 and 2 years. Most patients showed a stable clinical improvement to DBS and tolerated the treatment well. The authors of this study conclude that vALIC DBS for TRD showed continued efficacy in the long term (38).

In 2022, Bergfeld et al. reported the efficacy and quality of life after 6–9 years of DBS for depression in patients included in the RCT and reported in 2016 (41). The long-term follow-up of this study included 19 patients and 14 completed the study with a mean follow-up of 7.7 ± 1.5 years (41). The mean baseline HAM-D rating score was 22.2 ± 4.9 and at the last follow-up dropped to 12.0 ± 9.2. The study shows the continued efficacy of vALIC DBS in depression with sustained improvements in different aspects of quality of life assessed using the World Health Organization Quality of Life Assessment (WHOQOL-BREF) (41). After reporting the clinical outcomes of the above mentioned RCTs regarding VC/VS or vALIC DBS, the long-term observation of up to nearly 7.5 years in both studies showed sustained and meaningful improvement of depressive symptoms (37, 41).

The total number of patients with TRD treated by NAc/VS, VC, or vALIC is estimated to be 85 individuals worldwide. The response rate to DBS defined as a 50% reduction of MARDR scores was achieved in 39.8% of the patients (range, 23–53%). The remission defined as MADRS scores of < 10 was found in 17.8% after DBS (range, 0–40%). The mean follow-up was 19.7 months (range 3.7–24 months).

## Complications related to DBS procedures for TRD

Complications of DBS procedures can be grouped into three categories, primary surgery-related, hardware-related, and stimulation-induced complications. The surgery-related complications due to NAc/VS or VC DBS were minor, usually transient, and without a profound impact on the affected patients' health (29, 36–39, 41).

Moreover, the hardware-related complications were less common than stimulation-induced adverse events in a group of patients treated by NAc/VS or VC DBS for TRD (29, 39, 41). The most common complications were stimulation-related and resulted in hypomanic or manic states (31–34). These mood-related complications seen in an early postoperative period are possibly related to the overstimulation of the NAc/VS or VC—the main structures of a reward circuit. Rather a slow adjustment of stimulation parameters in these reward circuit structures may contribute to less stimulation-induced mood changes (30, 31, 36–39, 41). This relatively high incidence of stimulation-induced mood changes has been shown clearly in the first RCT provided by Dougherty et al. in the early postoperative blinded phase (36). Mood changes (e.g., insomnia, hypomania, disinhibition, suicidal ideation, and irritability) were mostly or only seen in the active but not in the sham-stimulation group (36). When compared to the stimulation parameters used for the control of movement disorders, the stimulation parameters used for TRD are relatively high, especially the pulse width and stimulation amplitude (30, 31, 35–39, 41).

The other relatively common adverse events of NAc/VS and VC DBS for TRD are suicidal thoughts, suicidal attempts, and suicides during the follow-up period (29–31, 36–39, 41). The authors of these studies did not consider these suicidal events to be related to the DBS treatment (30, 31, 36–39, 41). The patients treated for TRD by DBS constitute a vulnerable group of individuals and a close follow-up is mandatory to reduce such fatalities as attempted suicides or suicidal behaviors. The patients who fail to respond to DBS may be put at increased risk of suicidal thoughts and suicidal attempts during the follow-up period. The inclusion criteria for TRD trials using DBS should be redefined, paying more attention even to the patients with a good response that also does not preclude suicidal attempts after DBS. The long-term follow-up of VC/VS DBS studies has shown that suicidal ideation or suicide attempts are decreased over the follow-up months, indicating a stabilization of suicide risk in the long term (36–39, 41).

In the first clinical series presented by Bewernick et al., one patient developed psychosis, two patients had hypomania, and one patient committed suicide (29, 30). In the RCT of VC/VS DBS, there were the following complications: one patient committed suicide and three patients developed hypomanic or manic states without previous history of bipolar disorder (36). Another RCT of vALIC for TRD reported five suicides not clearly linked to stimulation among 25 individuals who entered the study (41). Other adverse events included two patients with mania and one patient with hypomania. Moreover, two patients withdrew from the study (37). Both of these patients after DBS discontinuation died shortly afterward (one patient committed suicide and the other had legal euthanasia). Taking into consideration the above mentioned serious adverse events, special attention to slow titration of stimulation parameters with careful expertise monitoring of the psychical state of operated patients is mandatory.

To sum up, among 85 patients included in this analysis treated by NAc/VS or VC DBS for DTR, there were 6 (7%) patients affected by suicidal thoughts, 12 (14%) patients developed suicidal attempts, and 4 (4.7%) patients committed suicide. The stimulation-induced adverse events including hypomania affected 9 (10.5 %) patients, mania was diagnosed in 5 (5.8 %) patients, and 2 (2.3 %) patients were diagnosed with psychosis/disinhibition. These adverse events related to suicidal attempts/thoughts and suicide as well as hypomanic and manic states warrants further scrutinized evaluation and assessment in future studies of NAc/VS and VC DBS for TRD.

## Limitations of current DBS studies for TRD

The clinical trials of NAc/VS and VC DBS for TRD have delivered evidence of clinical efficacy (29, 30, 32, 33). There are a lot of factors that make it difficult to compare the NAc/VS and VC DBS clinical trials for TRD. The researchers used incompatible inclusion and exclusion criteria, set different stimulation parameters in an early postoperative period, and various study designs impact the final outcome. Moreover, most of the studies are open-label, with a limited number of individuals included.

The study design may have a profound impact on the final outcomes. This situation is clearly visible in two RCTs of VC/VS and vALIC DBS for TRD (36, 37). The first trial of VC/VS DBS that failed to meet its primary endpoint began with up-front randomization followed by open-label treatment (36). The second trial of vALIC followed the opposite manner of the study design—the first open-label optimization for a relatively extended time period to allow the stimulation adjustment followed by randomization (37). Designing clinical trials of DBS for TRD must take into account several factors that may greatly impact the final outcomes. Trials that randomize patients to sham vs. active stimulation must make each arm long enough in duration to allow clinical differences to emerge. The preview duration of each arm as minimum time is regarded as 3 months. A too-short randomization period may result in the lack of clinical difference observed between sham and stimulation arms as observed in the recent DBS trials for TRD (36).

Another important question is whether to make the up-front randomization or randomize after an open-label optimization period. Both strategies of designing a trial of DBS for TRD have limitations (36, 37). With up-front randomization, there is often a limited time period to adjust the stimulation parameters (30). Finding the optimized stimulation parameters in individual patients is time consuming. This situation is complicated, but the fact is that the experience of NAc/VS or VC DBS for TRD is still limited (29, 36–39, 41). The up-front randomization carries the risk of comparing no optimized active stimulation to sham, potentially reducing the differences between the randomized groups (36). This situation is further obscured by the placebo effect in a sham group ranging from 10% to even 20%. Moreover, intense clinical attention may have a positive curative impact on the patient's health. The improvement in a sham group may be attributable to a microlesional effect seen after the surgery. The inclusion of a delay period after the surgery estimated at 2–3 months after the microlesional effect subsides may help partially alleviate the sham-related effects.

The above mentioned concerns related to the up-front optimization have led some authors to perform the first open-label optimization period followed by randomization for TRD (37). During the optimization period, patients can offer to discern if the stimulation is off or on after experiencing its effects over several months. This situation with turning the stimulation off may automatically unblind the patient. Another factor related to the optimization period is the so-called nocebo effect, in which the patient may worsen given the prospect of being turned off during the randomization period. The nocebo effect may influence both groups—the stimulation and the sham one, but in the stimulation group, it could produce worsened symptoms, despite active stimulation.

The above mentioned drawbacks of a trial may shed light on future RCT trials for TRD with a randomized double-blind crossover sham design. First of all, the optimization period after DBS should be long enough to assess the effects of DBS therapy for TRD symptoms and it should last at least 6 months or more. A longer optimization phase may also reduce a placebo effect, which is very strong in most DBS clinical trials for psychiatric indications. The clinical nature of TRD should be taken into account with its waxing and waning clinical symptoms (1–4). Longer follow-up periods enable the determination of more convenient stimulation settings, which may be specific to both targets (36, 37). This situation is clearly reflected in the extended period or maintenance period of RCTs for TRD (38). In the observations of some authors, the effects of DBS on TRD may not be visible up to 6 years after DBS procedures (41). This time period is extremely long when compared to the visible effects of DBS on motor symptoms in essential tremor and Parkinson's disease. Another problem related to so long follow-up period is the dropout of the patients initially included in both RCTs for TRD (36–39, 41). The Reclaim study included 30 patients treated by bilateral VC/VS for TRD while the long-term open-label phase presented by Hitti et al. covered only eight patients (36, 39). In the Bergfeld study published in 2022, only 14 patients from an initial number of 25 patients completed the long-term follow-up period (41).

A limitation factor, which is currently often forgotten, is the implementation of different neurosurgical techniques during DBS leads placement by various surgical teams (29, 36–39, 41). This factor is related to the use of intraoperative microrecording, macrostimulation, and awake or asleep procedures during DBS procedures (29, 36–39, 41).

Another factor is the visible difference in stereotactic coordinates between both RCTs for TRD (36, 38). This is related to the fact that in the Reclaim study, the stereotactic target was chosen more anterior and ventral (VC/VS) when compared with Bergfeld's study (36, 39). The target in Bergfeld et al. study was vALIC (37, 41). The initial stimulation settings in both studies differed considerably making direct comparison of clinical outcomes more difficult (36, 37).

Nowadays, TRD is regarded as a neuronal connectivity disorder. It has been shown that resting-state functional connectivity predicts the success of DBS of distinct anatomical targets (42). The success of a DBS procedure may be more related to the engagement of specific neuronal fibers running through the VC/VS and neuronal circuits than relying on anatomical coordinates (36, 39, 41). This situation confirms the belief that depression is a disorder of neuronal brain circuits, and effective stimulation depends to the greatest extent on the modulation of fibers connecting the areas of the brain that are responsible for the pathophysiology of depression (40, 42–45).

## Conclusion

The NAc/VS and VC represent two of the several targets being explored as a therapy for TRD. The open-label studies of NAc/VS and VC DBS for TRD have shown very promising results, but these results were tempered by RCTs outcomes. The further follow-up of TRD patients initially included in RCTs has shown a meaningful and sustained clinical benefit from DBS. The extension of both RCTs has shown that this clinical improvement is maintained for up to 7.5 years after surgery (38, 39, 41). Evaluating the outcomes of both these RCTs for TRD must be regarded with great caution due to the relatively high dropout numbers of patients in both studies. (38, 39, 41). Direct comparison of clinical outcomes of both studies is impossible due to many variables like differences in stereotactic targets, different initial stimulation settings, and clinical rating scales assessing depressive symptoms (36–38, 41).

The limited experience worldwide regarding DBS for TRD causes this treatment modality to be still regarded as experimental. Further trials are required to determine many factors such as stimulation settings, mode of stimulation, and patients' population for which DBS would be effective. The modern technologies incorporated in surgical planning such as tractography may enhance the clinical outcomes. The functional imaging on distinct brain activity using positron emission tomography may shed some light on DBS effects on TRD symptomatology. The future clinical trials should be long enough to permit the observation in years of TRD patients. This long-lasting observational approach of DBS in TRD patients may revolve around the true impact of this usually life-long psychiatric disorder. Moreover, the operated individuals with TRD are a very vulnerable group of patients. The close postoperative follow-up is mandatory to prevent exacerbation of manic or depressive episodes which may culminate in suicidal attempts. Therefore, such DBS studies for TRD should only be administered in clinical settings driven by experienced multidisciplinary teams.

## Data availability statement

The original contributions presented in the study are included in the article/supplementary material, further inquiries can be directed to the corresponding author.

## Author contributions

MS, MP, and TP contributed to conception, design of the study, and wrote sections of the manuscript. MS and MP organized the database and performed literature search with statistical analysis. All authors contributed to the creation of manuscript and revision of the manuscript. All authors read and approved the submitted version.
